# A study on why foreign-born East Asians but not US-born East Asians are underrepresented in leadership attainment in the U.S.

**DOI:** 10.1038/s41598-024-58342-x

**Published:** 2024-04-19

**Authors:** Jing Cao, Song Zhang

**Affiliations:** 1 Department of Statistics and Data Science, Southern Methodist University, Dallas, USA; 2 UT Southwestern Medical Center, Peter O’Donnell Jr. School of Public Health, Dallas, USA

**Keywords:** Psychology, Environmental social sciences

## Abstract

A recent study investigated the impact of culture of Asian groups on leadership attainment in the U.S. It revealed that East Asians (EAs) are less likely than South Asians (SAs) and white people (WP) to attain leadership positions, and SAs may even surpass WP in leadership attainment. The study explained that the underrepresentation of EAs in leadership positions in the U.S. (the so called bamboo ceiling) is partly because EAs communicate less assertively. Specifically, EA cultures value collectivism (e.g., humility and harmony), which are at odds with western cultures that value individualism (e.g., extraversion and assertiveness), whereas SA cultures are congruent with western cultures. However, the study did not distinguish the different impact of home culture (i.e., EA cultures) and host culture (i.e., western cultures) on US-born EAs versus foreign-born EAs. We argue that for US-born EAs (i.e., second generation EAs), host culture plays a more important role than home culture in their growth and they may not be underrepresented in leadership attainment compared to WP. The bamboo ceiling effect is mostly demonstrated among foreign-born EAs (i.e., first generation EAs) who are shaped mainly under the home culture. We support the argument by conducting analysis on one of the datasets in the original study and a new dataset from Fortune’s 40-under-40 list. Our study suggests that when studying the underrepresentation of leadership attainment for EAs, US-born EAs and foreign-born EAs should not be aggregated in one category. Considering the ethnic EA group, the bamboo ceiling phenomenon may exist mainly among foreign-born EAs because US-born EAs, with a median age of 21.3, are much younger than the general American population, who may not be experienced enough to be considered for leadership positions in established large companies.

## Introduction

In the United States, the Asian population rose to a record 22 million in 2021, and it is projected to surpass 46 million by 2060^1^. Based on measures of economic wellbeing, such as education, household income, and unemployment rate, Asian Americans do better than the overall U.S. population on average, but with large variation among different Asian origin groups^2,3^. Yet success in the economic wellbeing status of Asians obscures the fact that they are underrepresented in leadership positions, which has been described as the “bamboo ceiling”^4,5^. The term was coined by Asian American career coach Jane Hyun in her 2005 book “Breaking the Bamboo Ceiling: Career Strategies for Asians”. It is a derivative of the metaphor of glass ceiling which refers to invisible barriers in professional advancement for certain groups such as women and members of minorities. A number of works based on qualitative study have been conducted to investigate the leadership experiences of Asian Americans^6–8^. Whereas most studies grouped all Asians together, the work by Lu et al.^9^ recognized the impactful cultural differences between East Asians (e.g., ethnic Chinese, Japanese, and Koreans) and South Asians (e.g., ethnic Indians and Pakistanis), the two largest Asian groups in US. It represents one of the first systematic data-driven examinations of the mechanisms and scope of the bamboo ceiling phenomenon among East Asians (EAs) and South Asians (SAs). Based on an impressive set of nine studies (n = 11,030), the authors provided evidence that 1) EAs were less likely than SAs and white people (WP) to attain leadership positions and that SAs actually outperformed WP (i.e., EAs, not SAs, suffered from the bamboo ceiling); and 2) among a number of factors considered (e.g., socioeconomic status, personality traits, age, and gender), the cultural difference in assertiveness is consistently associated with the leadership attainment differential between EAs and SAs. The work by Lu et al.^9^ is closely related to the social identity theory of leadership which investigates the association between leadership and the social psychology of influence. It claims that group prototypical leaders are better supported by group members (due to their conformation to the mainstream culture) than less prototypical leaders^38,39^.

Between SA cultures and EA cultures, SA cultures encourage active interpersonal communication, argumentation, and debate^10^. For example, Indians like to converse and engage in animated discussions, and they are very people oriented^11^. The communication and networking styles in SA cultures are consistent with those traits embedded in the American prototype of leadership, which values confidence, assertiveness, and interpersonal skills. EA cultures, on the other hand, are under the enduring influence of Confucianism, which value humility, conformity, and deference to authority, where outspokenness and questioning of authority are strongly discouraged and viewed as disturbing factors of harmony^14,15^. EA cultures do not encourage extraversion; rather, they emphasize and respect qualities such as being reflective, reserved, and unassertive^8,16,17^. In contrast to SA cultures, EA cultures are at odds with the mainstream dynamic of assertiveness and directness in the U.S.

Lu et al.^9^ included whether a participant in the study was US born (i.e., US-born) as one of the control variables in their models. It was incorporated as a single additive term, which assumes that US-born has the same effect on all the ethnic groups. Among the models constructed on 5 datasets, US-born has shown a positive effect on leadership attainment in 3 models with an insignificant effect in the other 2 models. The authors didn’t investigate whether US-born EAs and foreign-born EAs differed in leadership attainment, and they concluded EAs as one group were underrepresented in leadership attainment. In this paper, we test a different hypothesis: US-born EAs were not underrepresented in leadership attainment, it is foreign-born EAs who were more likely to hit the bamboo ceiling. The reason behind this is that mainstream American culture generally plays a more important role for US-born EAs in their growth than EA cultures, and vice versa for foreign-born EAs.

In order to shed light on why we formulate such a hypothesis, we first need to understand the history of Asian immigration into the U.S. The passage of the 1965 Immigration and Nationality Act removed barriers for non-European immigration to the U.S. and created temporary worker programs for skilled workers, leading to an increase in the number of immigrants from Asian countries in the U.S.^18^. Among Asian Americans, about 30% trace their roots to East Asia (e.g., China, Japan, Korea) and 30% to South Asia (e.g., India, Pakistan, Bangladesh), with China (20.2%, including Taiwan) and India (19.1%) being the countries with by far the largest number of Asian immigrants^1^.

Between 1965 and 1976, the Chinese communist party and the American government were in a diplomatic freeze, so that EA immigrants in that period were mainly from Japan, Taiwan, and South Korea. Two years after President Nixon’s 1976 visit to mainland China, China started to relax its emigration control, and the U.S. – China diplomatic relationship was normalized in 1979. The number of immigrants from mainland China to the U.S. nearly doubled from 299,000 in 1980 to 536,000 by 1990, and again to 989,000 by 2000, reaching 2.5 million by 2018^19^.

Since most Asian Americans arrived in the U.S. after 1965, the populations of foreign-born Asian Americans (i.e., first generation Asian Americans) and US-born Asian Americans are unbalanced. About 57% of Asian Americans, including 71% of Asian American adults, were born outside of the U.S.^1^. As of 2019, the median age of the nation’s population was 38.4, whereas that of foreign-born EAs was 46.7, and US-born EAs was just 21.3^20–22^. In general, leadership positions favor senior people: the average age for a C-suite member (CEO, CFO, CHRO, CMO, CIO/CTO) is 56^23^. CEOs under 50 are a rare find in the S&P 500 companies^24^. Most EAs over the age of 50 are foreign-born; consequently, culture of their heritage plays a major role in molding their personalities. It is reasonable to assume that most US-born EAs, who grew up in western cultures, are not yet experienced enough, due to their younger age, to be considered for leadership positions in established large companies.

In light of the information on the age imbalance of US-born EAs and foreign-born EAs, we investigate which of the two cultures, home culture (i.e., heritage culture) or host culture (i.e., mainstream culture), has a stronger impact on leadership attainment for EAs. Home culture refers to the initial influence of culture inherited from ethnicity/parents, while host culture refers to the culture of the country where a person is currently living. They are widely adopted to study acculturation among international immigrants and their immediate descendants^40,41^. In the U.S., children grow up in a culture that values individualism, where they are taught to attend to the self and to understand the importance of asserting the self^16^. In EA countries, children grow up in a culture that values collectivism, where they are taught to fit in with others and to understand the importance of harmonious interdependence^16^. For EAs, their home culture and host culture differ dramatically. Perceived to have been assimilated and acculturated into mainstream American culture and not to conform to EA cultures, US-born EAs are sometimes called “banana”, i.e., “yellow on the outside, white on the inside”^25^.

Using one of the datasets published by Lu et al.^9^ and a new dataset based on Fortune’s 40-under-40 list the authors collected, we have studied the different impact of home culture and host culture on EAs in their leadership attainment. There is a rich collection of literature on acculturation which suggests that migrants and their second generations face a tension between embracing the cultures of their home and/or host countries^40–43^. In this study, we use birth-place as a proxy for culture identity. It assumes that US-born Asians are more likely to embrace American culture due to their upbringing and exposure to American society, while foreign-born Asians receive more influence from their home culture to shape their behaviors and identities. Following this assumption, we conduct studies to show that for US-born EAs, host culture plays a more important role than home culture in their growth and they were not underrepresented in leadership attainment compared to WP. It is foreign-born EAs who were more likely to hit the bamboo ceiling because they were shaped mainly under the home culture. This implies that not only are there significant cultural differences among Asian ethnic groups, there are significant culture differences (home culture versus host culture) among EAs. When it comes to studies that are associated with cultures, foreign-born EAs and US-born EAs should not be aggregated into a monolithic group .

## Study 1

The first dataset consists of data collected from Fortune’s 40-under-40 list (https://fortune.com/40-under-40/). The list includes individuals under the age of 40 considered by Fortune magazine to be the most influential young leaders for the year. Compared to the data on ethnicity of CEOs in the S&P 500 companies which was included in Lu et al.^9^, we chose this data source because it is more favorable to US-born Asian Americans who are relatively younger. Since the sample size is small for each year, we aggregated the data into two cohorts to reduce random variation. The first cohort contains data from the first 5 years of the archive (2009–2013), and the second contains data from the more recent 5 years (2017–2021). Together, the two cohorts provide information on the change of leadership attainment over the last decade. In this study, we focused on EAs and SAs who have made the list. To reduce confounding factors, we only included EAs and SAs on the list that attained leadership in the U.S., excluding those who have inherited a family business or developed their career in their country of origin (*SI Appendix,* Table S1). We gathered information on two variables: (1) whether or not the person was born in the U.S.; and (2) whether he or she received an undergraduate education in the U.S. or another western country (such as in Britain). Fortune’s 40-under-40 list only includes information on those leaders’ career development, without information on their biographies. In addition, many of the young leaders don’t have easily accessible detailed biographies. Therefore, we collected the data for the variables above from a systematic review of the following public sources: Wikipedia, LinkedIn, and internet-accessible interviews. A summary of these data is shown in Table 1.

**Table 1:** Statistics of the EA and SA leaders on Fortune’s 40-under-40 list.

Period	Total No.	No. EA	No. EA: US_Born	No. EA: US_educ	No. SA	No. SA: US_Born	No. SA: US_educ
2009–2013	245	6	5	6	7	4	7
2017–2021	405	19	10	17	23	13	21

In 2020, the list includes 40 influential people in each of five categories: finance, technology, healthcare, government and politics, and media and entertainment, which is why the 2017–2021 cohort has more people.

Several observations can be made based on Table 1. First, there are similar numbers of EAs and SAs in each cohort (6 EAs and 7 SAs in the 2009–2013 cohort, and 19 EAs and 23 SAs in the 2017–2021 cohort). Second, the percentage of EAs (4.7%) and SAs (5.7%) appearing on Fortune’s list in the 2017–2021 cohort was nearly double that of the 2009–2013 cohort (EAs: 2.4%; SAs: 2.9%). Third, in the 2009–2013 cohort, 100% of EAs and SAs received their undergraduate education in the U.S. or in a western country; in the 2017–2021 cohort, about 90% of them did.

The main hypothesis based on this study is that in each cohort there is little difference among EAs and SAs to be selected in the Fortune’s 40-under-40 list given they had been exposed to the host culture at an early age (i.e., being born in U.S. or having received undergraduate education in a western country). In the 2009–2013 cohort, among the 9 US-born Asians, 5 are EAs and 4 are SAs, the p-value for testing the null hypothesis (i.e., the proportion for EAs among EAs and SAs equals to 0.5 under a one-sample proportion test) is 0.74. In the 2017–2021 cohort, among the 23 US-born Asians, 10 are EAs and 13 are SAs, the *p*-value is 0.53. The results stay the same if we choose the condition as having received a western undergraduate education, where the p-values are 0.78 and 0.51, respectively. Thus, the data shows strong evidence that given EAs and SAs had been exposed to the host culture at an early age, there is little difference among them in leadership attainment in the U.S. The second hypothesis is that the proportion of Asians selected in the 40-under-40 list has increased in the second cohort. There are 13 Asians among the 245 leaders selected in the the 2009–2013 cohort, 42 among 405 in the 2017–2021 cohort. The p-value for the test (i.e., the proportions of Asians in the two cohorts being the same under a two-sample proportion test) is 0.017, lending evidence to the second hypothesis. This trend may result from the fact that more EAs and SAs who were mainly influenced by the host culture came to be mature enough to step into the leadership role in the second cohort. The two hypothesis tests together support the claim that when EAs were exposed to the host culture at an early age and if they were given enough time to grow and get mature, they were not underrepresented in leadership attainment.

## Study 2

This study is based on the dataset in Study 4 in Lu et al.^9^, which was conducted to examine leadership attainment of EA (n = 292), SA (n = 149), and white (n = 765) students in a group of 1,523 MBA students at a top business school. Readers may refer to Lu et al.^9^ for more details. The outcome variable is leadership nomination which is based on a survey asking students to nominate leaders in their class. Each student can submit 1–5 nominations. The explanatory variables include age, gender, socioeconomic status (ESE), the Big Five personality traits (openness to experience, conscientiousness, extraversion, agreeableness, and emotional stability), assertiveness, and whether a participant was born in the U.S.

**Figure 1:**
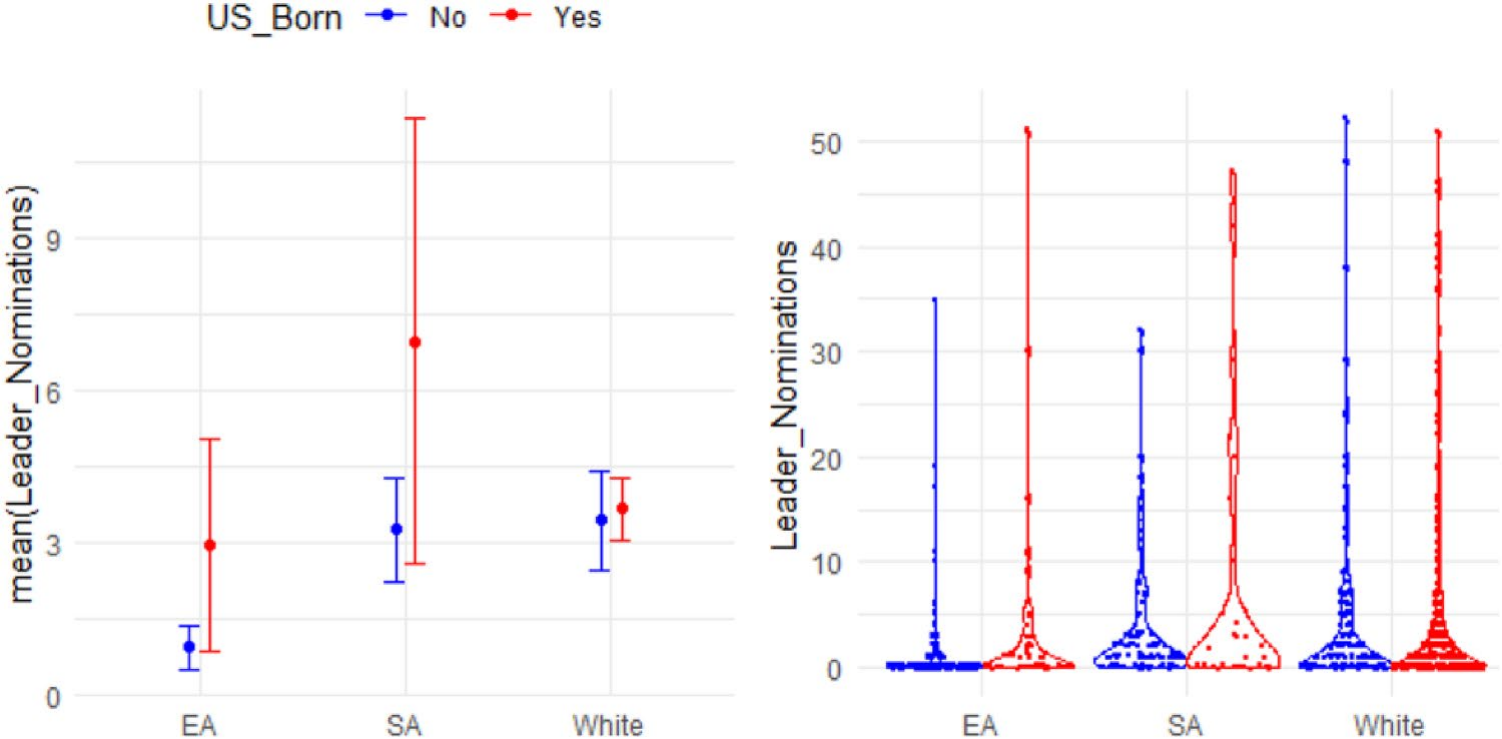
Comparison of leadership nomination between the US-born group and the foreign-born group for EAs, SAs, and WP in Study 2.

Figure 1 shows the comparison of leadership nomination (LN) between the US-born group and the foreign-born group for EAs, SAs, and WP, respectively. The left plot shows the 95% CI for the mean of LN, and the plot on the right shows the distribution of the observed values of LN. US-born EAs (M = 2.95 LNs, SD = 7.93) were significantly more likely to be nominated as leaders than foreign-born EAs (M = 0.94 LNs, SD = 3.33, t = 1.90, *P* = 0.03, based on the two sample t test). The difference in LN is smaller for the two groups of SAs (US-born SAs: M = 7.09 LNs, SD = 12.27; foreign-born SAs: M = 3.25 LNs, SD = 5.54; t = 1.72, *P* = 0.05) and barely noticeable for WP (US-born WP: M = 3.66 LNs, SD = 7.35; foreign-born WP: M = 3.46 LNs, SD = 7.10; t = 0.35, *P* = 0.36). This means that US-born does not have the same influence on LN for different ethnicity groups.

Lu et al.^9^ constructed a multilevel Poisson regression model to predict LN that included all the explanatory variables (*SI Appendix,* Table S2), where the dummy variable US-born was treated as an additive term with no interaction with any other independent variables. This model assumes that the effect of a U.S. birthplace is the same for EAs, SAs, and WP. Based on the evidence that US-born may affect LN differently for the different ethnicity groups, we extended the model to include interaction terms between US-born and the other covariates (*SI Appendix,* Table S3). The model comparison based on AIC and BIC (Model 4 in Table S2: AIC = 8586.01, BIC = 8657.11 vs. Model 4 in Table S3 : AIC = 8482.34, BIC = 8609.30) shows that the model fit of the interaction model is better than that of the additive model in Lu et al. (1). In addition, many of the interaction terms are statistically significant. Specifically, the interaction term between EA and US-born is significantly greater than 0 (B = 0.85, SE = 0.12, *P* < 0.001), indicating that the difference in LN between US-born EAs and foreign-born EAs is much larger than the difference between the US-born and foreign-born groups for SAs and WP.

Both Fig. 1 and the interaction model (*SI Appendix,* Table S3) show evidence that being US-born affects LN differently for EAs, SAs, and WP. To facilitate a more direct comparison, we fitted the Poisson regression model for the US-born group and the foreign-born group, separately, with the reference category for race as WP. The results are summarized in Table 2. When other variables are controlled, foreign-born EAs had substantially lower LN than WP (B =  − 0.73, SE = 0.08, *P* < 0.001), whereas US-born EAs had higher LN than WP (B = 0.14, SE = 0.08, *P* < 0.1). The models also show that foreign-born SAs had higher LN than WP (B = 0.21, SE = 0.07, *P* < 0.01), and US-born SAs had an even larger advantage in LN than WP (B = 0.48, SE = 0.07, *P* < 0.001).

**Table 2:** Multilevel poisson regressions predicting leadership nominations (Foreign-born vs. US-born).

	Model (Foreign-born)	Model (US-born)
Fixed effects		
Intercept	− 7.02***	− 5.90***
	(0.54)	(0.45)
White (reference category)		
East Asian	− 0.73***	0.14
	(0.08)	(0.08)
South Asian	0.21**	0.48***
	(0.07)	(0.07)
Age (years)	0.08***	0.10***
	(0.01)	(0.01)
Male	0.12	− 0.00
	(0.06)	(0.05)
Socioeconomic status	− 0.04*	− 0.05**
	(0.02)	(0.02)
Openness to experience	− 0.06*	0.04*
	(0.03)	(0.02)
Conscientiousness	0.13***	0.05*
	(0.03)	(0.02)
Extraversion	0.58***	0.53***
	(0.02)	(0.02)
Agreeableness	− 0.02	0.08***
	(0.03)	(0.02)
Emotional stability	− 0.08***	− 0.10***
	(0.02)	(0.02)
Assertiveness	0.51***	0.26***
	(0.06)	(0.04)
Random effects		
Intercept	0.00	0.05
	(0.00)	(0.22)
AIC	3102.97	5334.92
BIC	3159.00	5392.84
Log Likelihood	− 1538.48	− 2654.46

Standard errors of regression coefficients are in parentheses.

***p < 0.001; **p < 0.01; *p < 0.05.

**Figure 2:**
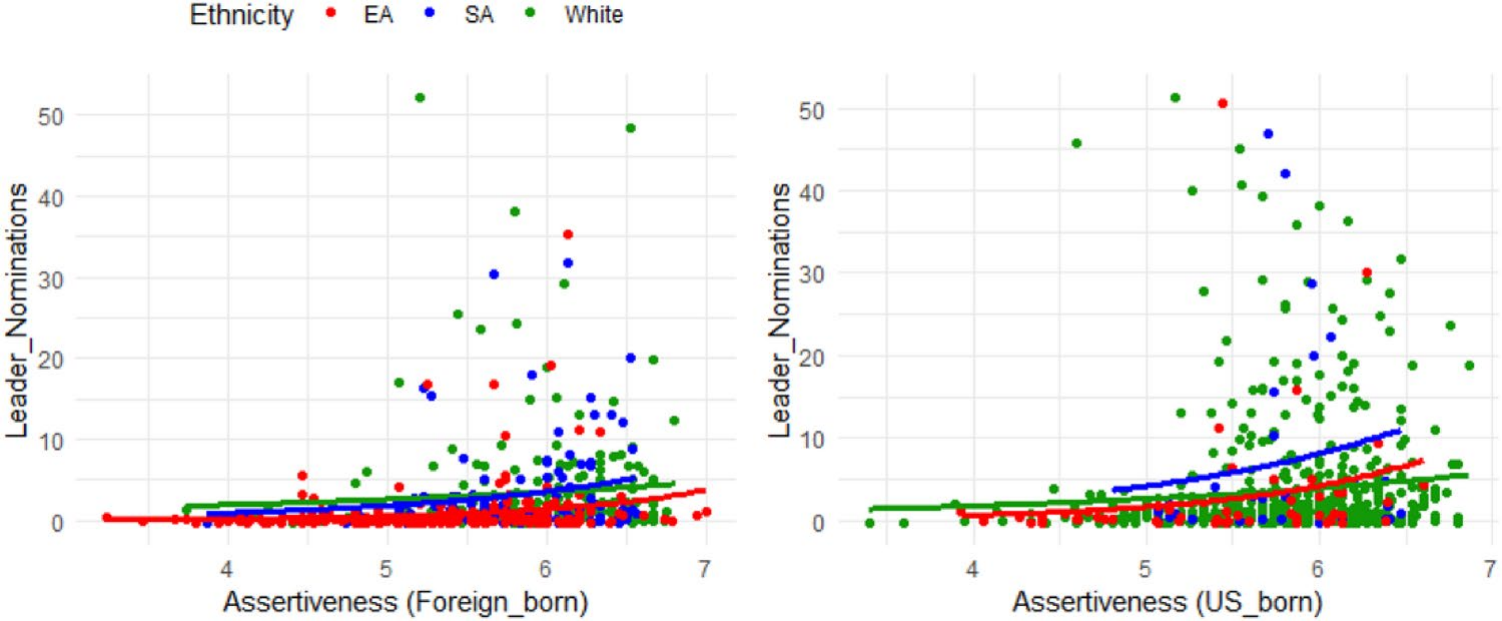
Leader nomination versus assertiveness for EAs, SAs, and WP in Study 2.

**Figure 3:**
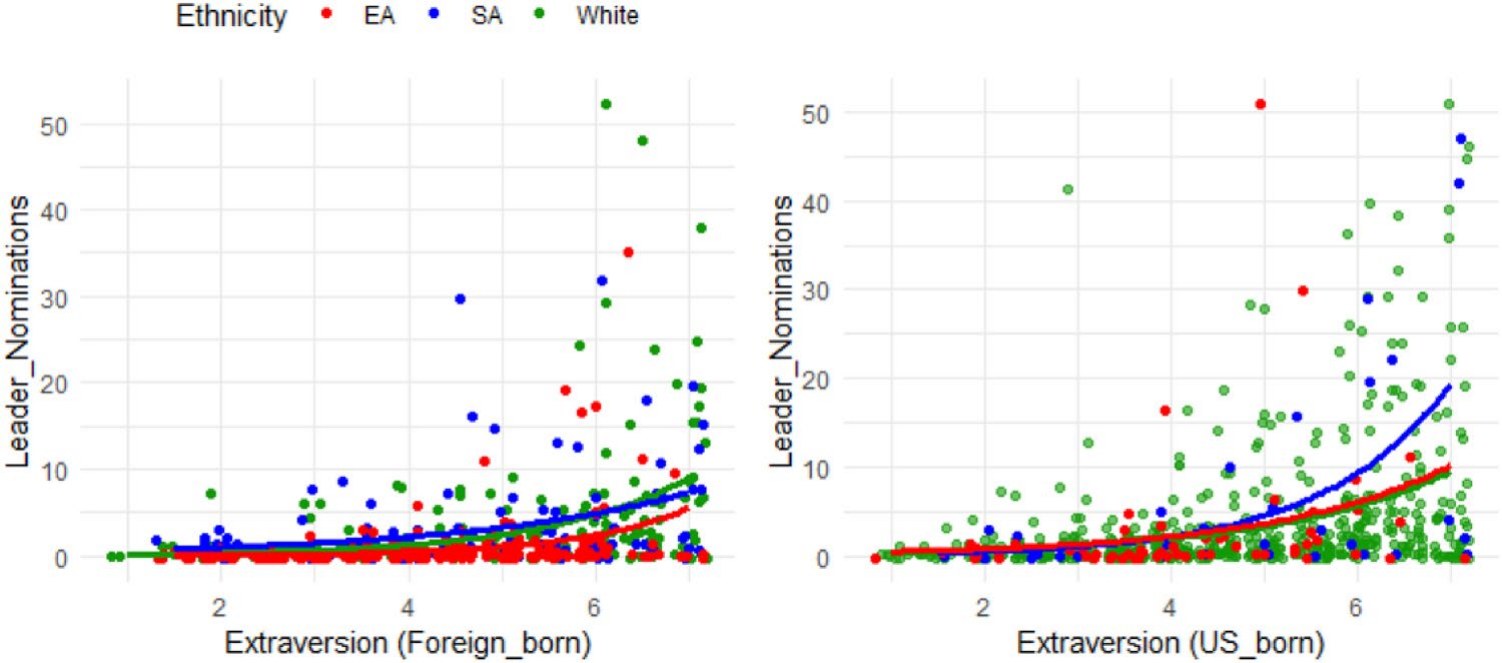
Leader nomination versus extraction for EAs, SAs, and WP in Study 2.

Among all the covariates (Table 2), assertiveness (US-born group: B = 0.26, SE = 0.04, *P* < 0.001; Foreign-born group: B = 0.51, SE = 0.06, *P* < 0.001) and extraversion (US-born group: B = 0.53, SE = 0.02, *P* < 0.001; Foreign-born group: B = 0.58, SE = 0.02, *P* < 0.001) increase the odds of LN the most for both the US-born group and the foreign-born group. Figures 2 and 3 illustrate their relationships with LN. For both of the birthplace groups, the figures show that a higher degree of assertiveness /extraversion is associated with higher LN across ethnicity groups.

Based on a reviewer’s suggestion, we also constructed a sequence of 4 models, with the results summarized in Table 3. Model 1 includes the non-cultural variables, Model 2 expands Model 1 by adding the ethnic categories (EAs, SAs, and WP; EAs as the reference category), Model 3 further includes the ethnic by US-born categories (EA/US-born, EA/foreign-born, SA/US-born, SA/foreign-born, WP/US-born, WP/foreign-born; EA/US-born as the reference category), finally Model 4 also adds extraversion and assertiveness. Model 2 shows that without including US-born (i.e., without distinguishing home culture and host culture), both SAs (B = 1.2, SE = 0.07, *P* < 0.001) and WP (B = 1.08, SE = 0.05, *P* < 0.001) significantly surpassed EAs in LN, demonstrating the general bamboo ceiling effect. Model 3 shows that US-born EAs had a significantly higher LN than foreign-born EAs (B =  − 1.27, SE = 0.1, *P* < 0.001), and their LN was not significantly different from those of foreign-born SAs (B = 0.1, SE = 0.09, *P* = 0.30) and WP (B = 0.12, SE = 0.09, *P* = 0.17). After adding extraversion and assertiveness, Model 4 shows that US-born EAs still had a significantly higher LN than foreign-born EAs. What is interesting is that US-born EAs had a significantly higher LN than US-born WP (B =  − 0.16, SE = 0.8, *P* < 0.05), foreign-born WP (B =  − 0.41, SE = 0.09, *P* < 0.001), and foreign-born SAs (B =  − 0.2, SE = 0.09, *P* < 0.05),. Though US-born EAs still had a significantly lower LN than US-born SAs, the difference is much smaller after controlling for extraversion and assertiveness (Model 3: B =  − 0.86, SE = 0.1; Model 4: B =  − 0.39, SE = 0.1). The AIC and BIC for Model 4 are both considerably smaller than the other three models, providing evidence for the validity of the comparison results.

**Table 3:** Multilevel poisson regressions predicting leadership nominations.

	Model 1	Model 2	Model 3	Model 4
(Intercept)	− 0.87***	− 1.89***	− 1.11***	− 5.34***
	(0.22)	(0.22)	(0.22)	(0.31)
Age	0.07***	0.09***	0.09***	0.09***
	(0.01)	(0.01)	(0.01)	(0.01)
Male	− 0.07*	− 0.14***	− 0.11**	− 0.05
	(0.03)	(0.03)	(0.03)	(0.04)
SES	0.00	− 0.03**	− 0.04**	− 0.04**
	(0.01)	(0.01)	(0.01)	(0.01)
East Asian (reference category)				
South Asian		1.20***		
		(0.07)		
White		1.08***		
		(0.05)		
EA/US-born (reference category)				
EA/Foreign-born			− 1.27***	− 1.19***
			(0.10)	(0.10)
SA/US-born			0.86***	0.39***
			(0.10)	(0.10)
SA/Foreign-born			0.10	− 0.20*
			(0.09)	(0.09)
WP/US-born			0.21**	− 0.16*
			(0.08)	(0.08)
WP/Foreign-born			0.12	− 0.41***
			(0.09)	(0.09)
Extraversion				0.54***
				(0.01)
Assert				0.31***
				(0.03)
Random effects				
Intercept	0.01	0.01	0.01	0.01
	(0.12)	(0.10)	(0.11)	(0.12)
AIC	11,550.40	11,005.50	10,790.36	8613.61
BIC	11,575.86	11,041.14	10,841.27	8674.63
Log likelihood	− 5770.20	− 5495.75	− 5385.18	− 4294.81

Unstandardized regression coefficients are displayed, with standard errors in parentheses.

***p < 0.001; **p < 0.01; *p < 0.05.

**Figure 4:**
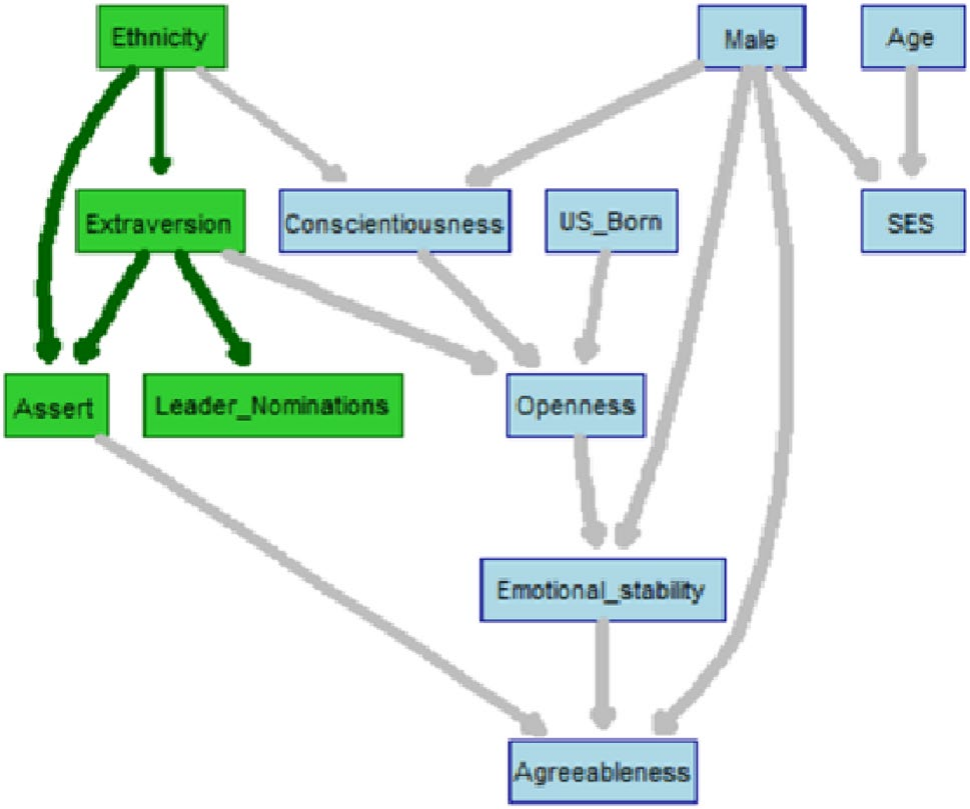
Bayesian network structure of the variables in Study 2.

We also conducted a Bayesian network (BN) analysis^26,27^ using the *bnlearn* package^28^ in R to describe and visualize the dependencies among all the variables in this dataset, where the relationships are not restricted to those specified by the parametric Poisson model. BNs are probabilistic graphical models, where variables are depicted as nodes and arcs represent probabilistic dependences among variables. They are capable of capturing the interplay of different variables and providing insight on how they influence each other, and have been adopted in areas such as psychology, medicine, and biology^29–31^. The final network structure was obtained by exploring and averaging 1000 network structures to reduce the impact of random local optimal network learning. The averaged network structure was based on choosing the arcs present in at least 85% of the 1000 networks explored. The resulting network structure shown in Fig. 4 highlights the pivotal relationships related to LN: given the other variables, ethnicity is directly associated with extraversion and assertiveness where extraversion is then linked to assertiveness and LN. This structure also agrees with the psychological theory that assertiveness is one of the facets of extraversion^32^.

The conclusion from Lu et al.^9^ based on the study is that EAs were less likely to be nominated as leaders than WP, while SAs were more likely to be nominated than WP, this effect was partially mediated by assertiveness. We re-analyzed the data and found that US-born EAs and foreign-born EAs had very different representation in LN and they should not be lumped in one group. Though foreign-born EAs were less likely to be nominated as leaders than WP, US-born EAs were actually more likely to be nominated as leaders than WP. Another conclusion is that assertiveness and extraversion are associated with people’s LN (both are highly significant in Model4, and adding them significantly improves model fit of Model 4 compared to Model 3), but they only partially mediate the LN gap between foreign-born EAs and the other groups. This is because the LN gap between foreign-born EAs and the other groups is reduced but not removed after assertiveness and extraversion are added in the model. This result was also demonstrated in Lu et al.^9^ There are other cultural aspects that contribute to the difference in leadership attainment for the ethnic groups. For example, Lu^44,45^ conducted studies to show that EAs are stereotyped as lacking creativity and socializing more with other EAs (i.e., ethnic homophily), both of them are at odds with prized leadership attributes of creativity and ethnic heterophily**.**

This study examined leadership attainment for young EAs and SAs in a business school whose average age was 27.9. Lu et al.^9^ also included two studies conducted to examine whether EAs are less likely than SAs to attain senior leadership positions in large US companies (data are proprietary and unavailable), where the participants’ mean age was around 40. In their Study 2, 858 EA employees and 867 SA employees from 18 S&P 500-level companies participated in the field survey, and in their Study 3a, 878 EA employees and 797 SA employees from another set of 16 S&P 500-level companies participated a year later. In both studies, leadership attainment was operationalized as whether or not a participant currently occupied an executive/senior leadership position (the data was missing for 51 EAs and 54 SAs in Study 2, and 9 EAs and 15 SAs in Study 3a). In their Study 2, the summary statistics on leadership attainment they provided were: US-born EAs (23.0%), foreign-born EAs (12.8%), US-born SAs (33.3%), and foreign-born SAs (29.3%). In Study 3a, the summary statistics were: US-born EAs (18.7%), foreign-born EAs (13.5%), US-born SAs (27.2%), and foreign-born SAs (24.7%).

This comparison of leadership attainment in senior leadership positions across large US companies reveals that 1) SAs are more likely than EAs to attain leadership in those companies; 2) for both EAs and SAs, being born in the U.S. will increase the chance of leadership attainment. The finding that is more relevant to our study is that being born in the U.S. has a bigger influence on EAs than on SAs in leadership attainment. Specifically, in Study 2, US-born EAs were 10.2% (23–12.8%) more likely than foreign-born EAs to attain leadership positions, the difference was 4% (33.3–29.3%) for SAs. In Study 3a, the difference was 5.2% (18.7–13.5%) for EAs and 2.5% (27.2–24.7%) for SAs. Taken together, the effect of being US-born versus foreign-born on leadership attainment for EAs is more than twice than that for SAs (10.2% vs. 4% in their Study 2, 5.2% vs. 2.5% in Study 3a). These summary statistics did lend some evidence that for older EAs and SAs who were in more advanced stage in their career, host culture has a stronger influence on leadership attainment for EAs than on SAs in the U.S.

## Discussion

Inspired by the study of Lu et al.^9^, we examined further why EAs seem to be less likely than SAs and WP to attain leadership positions in the U.S. Based on a large dataset in Lu et al.^9^ and a new dataset from Fortune’s 40-under-40 list, we used Poisson regression models, data visualization, and Bayesian network inference to draw the following conclusions. First, foreign-born EAs had significantly lower leadership attainment than WP, but US-born EAs did not follow the same pattern. One explanation is that host culture has a stronger impact for US-born EAs on leadership attainment than home culture. For foreign-born EAs, their ways of life were shaped by home culture which is at odds with western cultures. In contrast, for US-born EAs, their ways of life were mostly formed by host culture which constitutes the dominant part of their social interactions. They look different from WP, but they have been assimilated and acculturated into mainstream American culture which may explain why US-born EAs were not underrepresented in leadership attainment in the study.

We further argue that the bamboo ceiling suffered by EAs is at least partly due to the age imbalance between US-born EAs and foreign-born EAs. Since most EAs arrived in the U.S. after 1965, the median age of US-born EAs was only 21.3 in 2019, and the median age of foreign-born EAs was 46.7, where the median age of the US population was 38.4. Considering that 1) leadership positions usually favor more senior people and 2) immigrants from mainland China, which is the largest country in East Asia, only started to arrive in the U.S. in large numbers in the late 70 s, the majority of US-born EAs are in general not experienced enough to be considered for leadership positions in established large companies. On the other hand, based on the comparison of early-career leadership attainment which only considers young leaders, such as Fortune’s 40-under-40 list, we found that young EAs have become more competitive in leadership attainment in recent years, the percentage of EAs made to the list in the 2017–2021 cohort (4.7%) has almost doubled compared to that in the 2009–2013 cohort (2.4%).

Lu et al.^9^ suggested that EAs hit the bamboo ceiling partly because their low assertiveness is incongruent with the American prototype of leadership. This study found that both assertiveness and extraversion are positively associated with leadership attainment. Though the two factors are positively correlated, they differ enough that they both are incrementally predictive of leadership attainment. Extraversion is one of the five core traits of personality, which consists of several facets, including gregariousness, assertiveness, excitement-seeking, positive emotions, and warmth^32^. Numerous studies have shown that extraversion is associated with leadership performance^33–35^. Broadly put, the western cultures favor extraversion more and EA cultures favor extraversion less. Extraversion, among the five personality traits, are the strongest predictor of leadership attainment^36,37^. Extraverts are sociable, assertive, and energetic people. Compared to introverts who tend to be quiet, passive, and reserved, extraverts are more likely to take on leadership roles. The network structure produced by the Bayesian network analysis also confirmed the conclusion.

The study strengthens the theory that explains the bamboo ceiling phenomenon. Compared to the previous findings, it sheds light on the different impact of home culture and host culture on EAs in leadership attainment in the U.S. Many foreign-born EAs were heavily influenced by home culture which promotes values opposite to assertiveness and extraversion, they were not educated and trained in a way that is congruent with the western prototype of leaders. Thus they may have a disadvantage competing for leadership positions in the U.S. In contrast, US-born EAs, who grew up under host culture that promotes individualism and encourages competition for leadership, may not face a serious bamboo ceiling in leadership attainment. The observed bamboo ceiling phenomenon for EAs may stem from the fact that the majority of them are immigrants born in a country with differing cultural values. If this is true, then the leadership attainment gap between EAs and others will close as the current cohort of young EAs born in the U.S reach the zenith of their careers. Though this is a hypothesis based on this study, time will tell whether the hypothesis is correct or not.

### Supplementary Information

Supplementary Information.

## Data Availability

The dataset of the Fortune’s 40-under-40 list is included in the supplementary material.
