# Supplementary material for: A study on why foreign-born East Asians but not US-born East Asians are underrepresented in leadership attainment in the U.S
Source: Sci Rep. 2024 Apr 19;14:9015. doi: 10.1038/s41598-024-58342-x (PMC11031584; doi:10.1038/s41598-024-58342-x)
Supplement: Supplementary file 1 — Supplementary Information. [file 41598_2024_58342_MOESM1_ESM.docx]

**Supplementary Material**

Table S1. Study1: East Asian leaders and South Asian leaders in Fortune’s 40-under-40 list

| Cohort | # | East Asian Leader |  | # | South Asian Leader |
| --- | --- | --- | --- | --- | --- |
| 2009 - 2013 | 6 | Tony Hsieh  Wen Zhou  Perry Chen  Clara Shih  David Chang  Andrew Ng |  | 7 | Premal Shah  Salman Khan  Sid Sankaran  Raj Shah  Mona Mourshed  Hosain Rahman  Niraj Shah |
| 2017 - 2021 | 19 | \| Anthony Tan \| \| --- \| \| Tan Hooi Ling \| \| Sonia Cheng \| \| Feng Zhang \| \| Mei Mei Hu \| \| Carl Pei \| \| Peng Zhao \| \| Amy Hong \| \| Bill Pang \| \| Eric Wu \| \| Rick Yang \| \| Tony Xu \| \| Ya Xu \| \| Lily Peng \| \| Suelin Chen \| \| Yuh-Line Niou \| \| Bowen Yang \| \| Howie Liu \| \| Ed Yong \| |  | 23 | \| Leo Varadkar \| \| \| --- \| --- \| \| Divya Nag  Rishi Shah  Eila Janah  Shradha Agarwal \| \| \| Dhivya Suryadevara \| \| Anjali Sud \| \| Baiju Bhatt \| \| Anu Duggal \| \| Arjun Bansal \| \| Ankiti Bose \| \| Ahkhay Naheta \| \| \| Shivani Siroya \| \| \| Sunayna Tuteja \| \| \| Suneera Madhani \| \| \| Ambar Bhattacharyya \| \| \| Andrew Dudum \| \| \| Kartik Ramamoorthi \| \| \| Deepa Subramaniam \| \| \| Raj Kannappan \| \| Amit Paley \| \| Akila Raman \| \| Rohan Seth \| |

Note: The information such as birth country is not listed in the table to respect people’s privacy.

|  | **Model 1** | **Model 2** | **Model 3** | **Model 4** |
| --- | --- | --- | --- | --- |
| **Fixed Effects** |  |  |  |  |
| Intercept | 1.32^***^ | -1.00^***^ | -4.32^***^ | -6.48^***^ |
|  | (0.06) | (0.22) | (0.27) | (0.34) |
| **White** (reference category) |  |  |  |  |
| **East Asian** | **-0.98^***^** | **-0.88^***^** | **-0.49^***^** | **-0.38^***^** |
|  | **(0.05)** | **(0.06)** | **(0.06)** | **(0.06)** |
| **South Asian** | **0.10^*^** | **0.31^***^** | **0.32^***^** | **0.34^***^** |
|  | **(0.04)** | **(0.05)** | **(0.05)** | **(0.05)** |
| Age (years) |  | 0.09^***^ | 0.09^***^ | 0.09^***^ |
|  |  | (0.01) | (0.01) | (0.01) |
| Male |  | -0.12^***^ | 0.06 | 0.02 |
|  |  | (0.03) | (0.04) | (0.04) |
| **US born** |  | **0.37^***^** | **0.40^***^** | **0.40^***^** |
|  |  | **(0.04)** | **(0.04)** | **(0.04)** |
| Socioeconomic status |  | -0.04^***^ | -0.04^***^ | -0.05^***^ |
|  |  | (0.01) | (0.01) | (0.01) |
| Openness to experience |  |  | 0.02 | 0.02 |
|  |  |  | (0.02) | (0.02) |
| Conscientiousness |  |  | 0.05^**^ | 0.06^***^ |
|  |  |  | (0.02) | (0.02) |
| Extraversion |  |  | 0.57^***^ | 0.55^***^ |
|  |  |  | (0.01) | (0.01) |
| Agreeableness |  |  | 0.04^*^ | 0.06^***^ |
|  |  |  | (0.01) | (0.01) |
| Emotional stability |  |  | -0.07^***^ | -0.08^***^ |
|  |  |  | (0.01) | (0.01) |
| Assertiveness |  |  |  | 0.36^***^ |
|  |  |  |  | (0.03) |
| **Random Effects** |  |  |  |  |
| Intercept | 0.01 | 0.01 | 0.02 | 0.02 |
|  | (0.10) | (0.12) | (0.14) | (0.13) |
| AIC | 11230.17 | 10917.95 | 8706.82 | 8586.01 |
| BIC | 11250.53 | 10958.69 | 8772.88 | 8657.11 |
| Log Likelihood | -5611.08 | -5450.98 | -4340.41 | -4279.01 |
| ^Note. Unstandardized regression coefficients are displayed, with standard errors in parentheses^  ^***^p < 0.001; ^**^p < 0.01; ^*^p < 0.05 | | | | |

Table S2. Study 2: Multilevel Poisson Regressions Predicting Leadership Nominations in Lu et al. (1)

Table S3. Study2: Multilevel Poisson Regressions (with Interactions) Predicting Leadership Nominations

|  | **Model 1** | **Model 2** | **Model 3** | **Model 4** |
| --- | --- | --- | --- | --- |
| **Fixed Effects** |  |  |  |  |
| Intercept | 1.25^***^ | -0.79^*^ | -4.14^***^ | -7.02^***^ |
|  | (0.07) | (0.34) | (0.41) | (0.55) |
| **White** (reference category) |  |  |  |  |
| **East Asian** | **-1.27^***^** | **-1.37^***^** | **-0.89^***^** | **-0.71^***^** |
|  | **(0.08)** | **(0.08)** | **(0.08)** | **(0.08)** |
| **South Asian** | **-0.06** | **-0.03** | **0.14^*^** | **0.22^**^** |
|  | **(0.06)** | **(0.06)** | **(0.07)** | **(0.07)** |
| **US born** | **0.10^*^** | **-0.26** | **-0.10** | **1.08** |
|  | **(0.04)** | **(0.42)** | **(0.53)** | **(0.69)** |
| **East Asian * US born** | **1.05^***^** | **1.16^***^** | **0.98^***^** | **0.85^***^** |
|  | **(0.11)** | **(0.11)** | **(0.12)** | **(0.12)** |
| **South Asian* US born** | **0.68^***^** | **0.68^***^** | **0.34^***^** | **0.26^*^** |
|  | **(0.10)** | **(0.10)** | **(0.10)** | **(0.10)** |
| Age |  | 0.08^***^ | 0.08^***^ | 0.09^***^ |
|  |  | (0.01) | (0.01) | (0.01) |
| Male |  | -0.11 | 0.12 | 0.12 |
|  |  | (0.06) | (0.06) | (0.06) |
| Socioeconomic status |  | -0.02 | -0.03 | -0.04^*^ |
|  |  | (0.02) | (0.02) | (0.02) |
| Age* US born |  | 0.02 | 0.03^*^ | 0.02 |
|  |  | (0.01) | (0.01) | (0.01) |
| Male* US born |  | 0.00 | -0.08 | -0.12 |
|  |  | (0.07) | (0.08) | (0.08) |
| Socioeconomic status* US born |  | -0.03 | -0.02 | -0.01 |
|  |  | (0.02) | (0.03) | (0.03) |
| Openness to experience |  |  | -0.05 | -0.06^*^ |
|  |  |  | (0.03) | (0.03) |
| Conscientiousness |  |  | 0.13^***^ | 0.14^***^ |
|  |  |  | (0.03) | (0.03) |
| Extraversion |  |  | 0.60^***^ | 0.59^***^ |
|  |  |  | (0.02) | (0.02) |
| Agreeableness |  |  | -0.03 | -0.02 |
|  |  |  | (0.03) | (0.03) |
| Emotional stability |  |  | -0.05^*^ | -0.09^***^ |
|  |  |  | (0.02) | (0.02) |
| Openness to experience* US born |  |  | 0.09^*^ | 0.10^**^ |
|  |  |  | (0.03) | (0.03) |
|  |  |  |  |  |
|  |  |  |  |  |
| Conscientiousness* US born |  |  | -0.10^**^ | -0.10^**^ |
|  |  |  | (0.04) | (0.04) |
| Extraversion* US born |  |  | -0.05 | -0.06 |
|  |  |  | (0.03) | (0.03) |
| Agreeableness* US born |  |  | 0.09^**^ | 0.10^**^ |
|  |  |  | (0.03) | (0.03) |
| Emotional stability* US born |  |  | -0.04 | -0.01 |
|  |  |  | (0.03) | (0.03) |
| Assertiveness |  |  |  | 0.49^***^ |
|  |  |  |  | (0.06) |
| Assertiveness* US born |  |  |  | -0.22^**^ |
|  |  |  |  | (0.07) |
| **Random Effects** |  |  |  |  |
| Intercept | 0.01 | 0.01 | 0.02 | 0.02 |
|  | (0.11) | (0.11) | (0.13) | (0.12) |
| AIC | 11036.47 | 10790.09 | 8601.24 | 8482.34 |
| BIC | 11072.11 | 10856.29 | 8718.10 | 8609.30 |
| Log Likelihood | -5511.23 | -5382.05 | -4277.62 | -4216.17 |
| ^Note. Unstandardized regression coefficients are displayed, with standard errors in parentheses^  ^***^p < 0.001; ^**^p < 0.01; ^*^p < 0.05 | | | | |
